# First Report of Bovine Leukemia Virus Infection in Yaks (*Bos mutus*) in China

**DOI:** 10.1155/2016/9170167

**Published:** 2016-06-02

**Authors:** Jian-Gang Ma, Wen-Bin Zheng, Dong-Hui Zhou, Si-Yuan Qin, Ming-Yang Yin, Xing-Quan Zhu, Gui-Xue Hu

**Affiliations:** ^1^College of Animal Science and Technology, Jilin Agricultural University, Changchun, Jilin 130118, China; ^2^State Key Laboratory of Veterinary Etiological Biology, Key Laboratory of Veterinary Parasitology of Gansu Province, Lanzhou Veterinary Research Institute, Chinese Academy of Agricultural Sciences, Lanzhou, Gansu 730046, China; ^3^General Station for Surveillance of Wildlife Diseases & Wildlife Borne Diseases, State Forestry Administration (SFA), Shenyang 110034, China

## Abstract

Enzootic bovine leukosis (EBL) is a chronic lymphosarcoma disease of cattle caused by bovine leukemia virus (BLV). No information is available concerning the epidemiology of BLV infection in yaks (*Bos mutus*). One thousand five hundred and eighty-four serum samples from 610 black yaks and 974 white yaks from Gansu province, northwest China, were collected between April 2013 and March 2014 and tested for BLV antibodies using a commercially available ELISA kit. The overall BLV seroprevalence in yaks was 21.09% (334/1584), with 24.26% (148/610) black yaks and 19.10% (186/974) white yaks yielding positive results. Risk factor analysis indicated that with the exception of breed (OR = 1.36, 95% CI = 1.06–1.73, *P* < 0.05), the age, region, gender, farm, and the numbers of pregnancies were not considered as risk factors for the presence of BLV in yaks included in this study. This is the first report of BLV infection in yaks in China, which provides information for controlling BLV infection in yaks.

## 1. Introduction

Enzootic bovine leukosis (EBL) is a chronic lymphosarcoma disease of cattle caused by bovine leukemia virus (BLV), a member of the genus* Deltaretrovirus* in the family Retroviridae [1]. Transmission of BLV can occur through two routes: horizontal transmission (e.g., iatrogenic) and vertical transmission [2, 3]. BLV infections may present as aleukemic (asymptomatic animals), persistent lymphocytosis (with hematological abnormalities), and lymphosarcoma (tumors), and most infections are subclinical. Approximately 30% of BLV infected cattle have persistent lymphocytosis, and malignant lymphosarcoma occurs in less than 5% of infected animals [4, 5].

EBL can cause major economic losses to the cattle industry worldwide; it is listed by World Organization for Animal Health (OIE) as a disease of importance to international trade [6]. Many countries have investigated and reported the disease in cattle in recent years [7, 8], and the data have supported the prevention and control of EBL to reduce economic losses. However, only one study has reported the seroprevalence of BLV in cattle in northern and northeastern China [9].

Yaks are a unique bovine species and the precious semiwild animal species which lives at high altitudes. Most of them are distributed in the territory of China, especially the white yaks, which only live in the Tianzhu Tibetan Autonomous County (TTAC), Gansu province, northwest China. However, there was no information on the seroprevalence of BLV infection in yaks in China. Therefore, for the first time we surveyed the BLV seroprevalence in yaks in Gansu province, northwest China, and evaluated the risk factors that influenced BLV seroprevalence.

## 2. Materials and Methods

### 2.1. Study Area

Serum samples were collected from black and white yaks in various regions in Gansu province (32°31′′–42°57′′N, 92°13′′–108°46′′E), northwest China. These regions have a plateau continental climate, and the average annual temperatures vary between −8°C and 4°C.

### 2.2. Sample Collection

Blood samples were collected between April 2013 and March 2014. A total of 974 blood samples were collected from white yaks randomly from several farms in TTAC, Gansu province, northwest China. A total of 610 blood samples were collected from black yaks randomly from several farms in Luqu and Maqu counties, Gannan Tibetan Autonomous Prefecture, Gansu province, northwest China. All the blood samples were transported directly to the laboratory. Serum was obtained through centrifugation at 1000 ×g for 5 min. The serum was separated and stored at −20°C until analysis. Detailed information regarding breed, geographic origin, season, number of pregnancies, gender, age, and farm were recorded and listed in Tables 1 and 3.

### 2.3. Serological Assay

Serum samples were examined for antibodies against BLV using a commercially available ELISA kit (Pourquier, Montpellier, France) according to the manufacturer's instructions [10]. Positive and negative controls were provided with the kit. The optical density of color development was read by a photometer at 450 nm, and the titer of antibodies in the sera was determined following the manufacturer's recommendations [10].

### 2.4. Data Analysis

Seropositive yaks were analyzed in relation to gender, age, number of pregnancies, season, breed, and region. Exploratory analysis was performed to determine variables potentially associated with exposure to BLV. These factors were studied in a multivariable logistic regression model, and if the (*P*) value was <0.05, it was considered a risk factor for BLV infection. The statistical program PASW Statistics 19.0 (SPSS Inc., IBM Corporation, Somers, NY) was used to perform the statistical analysis.

## 3. Results and Discussion

It was demonstrated that 334 (21.1%) out of 1584 serum samples were BLV seropositive, with 24.26% in black yaks and 19.10% in white yaks (Table 1). The BLV seropositive yaks were found in all herds. BLV seroprevalence ranged from 18.63% to 25.1% in different age groups of yaks. The highest and lowest rates were recorded in the 3 < years < 5 and 5 < years old groups, respectively (Table 1). Male yaks had a higher seroprevalence (23.78%) compared to females (19.95%) (Table 1). Among yaks sampled in different seasons, the seroprevalence varied from 17.91% to 23.13% (Table 1). Among yaks from different regions, BLV seroprevalence ranged from 19.10% to 24.78% (Table 1). In addition, BLV seroprevalence in female yaks of different pregnancy status ranged from 18.57% to 20.11% (Table 1). Among the different farms, BLV seroprevalence varied from 16.00% to 28.16% (Table 3).

According to conditional forward stepwise logistic regression, it was found that gender, age, region, farm, and number of pregnancies of yaks were not significant risk factors for BLV (*P* > 0.05). The breed of yaks was considered to be a risk factor that influences the BLV seroprevalence in yaks (*P* < 0.05) (Table 2).

In this study, the overall BLV seroprevalence in yaks was 21.09%, which was slightly lower than that reported in dairy cattle (25.4%) in Iran [8] but higher than that in breeding beef (16.3%) in Japan [11]. The BLV seroprevalence in yaks in this study was much higher than previous reports [12, 13]. On the other hand, BLV seroprevalence in this study was much lower than the seroprevalence in cattle in Japan (28.6%) [11], in Ontario in Canada (29.4%) [14], and in Syria (69.2%) [15]. Many factors may be contributable to such differences, such as the climatic conditions, geographical conditions, species and farm management, and the sensitivity of the detection techniques used.

Once an animal is infected with BLV, it will remain infected for life. The prevalence determined for the different seasons therefore cannot be considered as a possible risk factor, although the BLV seroprevalence varied from 17.91% to 23.13% in different season groups, with slight differences among different seasons, and slightly higher seroprevalences were found in yaks sampled in spring (23.12%) and autumn (22.48%) (Table 1). This result was in line with the characteristic of BLV that BLV is a persistent infection.

Moreover, BLV seroprevalences in yaks of different age groups were slightly different, but the difference was not statistically significant (*P* > 0.05). The seroprevalence increased gradually from 19.93% in years ≤ 1 age group to 25.07% in 3 < years ≤ 5 age group, and the highest seroprevalence was the 3 < years ≤ 5 age group. These results are similar to that of a recent study [16]. These differences may be due to the fact that BLV incubation period is 3-4 years on average. Furthermore, BLV seroprevalences in yaks of different farm groups ranged from 16.00% to 28.16%. In general, the seroprevalence in farms of TTAC was slightly lower than in Maqu and Luqu counties. But it was not a significant risk factor for BLV (*P* > 0.05). These slight differences could be because TTAC is located near Lanzhou, the capital of Gansu province, and the yak farms are better managed than that in Maqu and Luqu counties.

This study showed that the breed of yaks was the only risk factor associated with BLV seroprevalence. Logistic regression analysis showed that the black yaks had a 1.36 times higher risk of being infected than the white yaks (OR = 1.36, 95% CI = 1.06–1.73). There could be a few explanations for this difference. First, white yaks and black yaks are different yak breeds, and they may have different susceptibility to infection with BLV due to their genetic difference. Second, white yaks get better animal welfare and are better managed because white yak is a unique and precious yak breed, only lives in TTAC, Gansu Province, Northwest China, and total number is only about 50000. They may have better resistance to infection with BLV than that of black yaks.

This is the first report of BLV seroprevalence in yaks in China. This study extended the host range for BLV. Moreover, the findings of the present study provided base-line information for the timely execution of strategies and measures to control BLV infection in yaks and to assess resulting effects.

## Figures and Tables

**Table 1:** Seroprevalence of bovine leukemia virus (BLV) infection in yaks in Gansu province, northwestern China, by enzyme-linked immunosorbent assay (ELISA).

Factor	Category	No. tested	No. positive	Prevalence (%)
Gender	Male	471	112	23.78
	Female	1113	222	19.95

Age (years)	0 < years ≤ 1	286	57	19.93
	1 < years ≤ 3	594	123	20.71
	3 < years ≤ 5	355	89	25.07
	5 < years	349	65	18.63

Pregnancy	≤2	854	171	20.02
	3–5	189	38	20.11
	≥6	70	13	18.57

Season	Spring	428	99	23.13
	Summer	354	70	19.77
	Autumn	467	105	22.48
	Winter	335	60	17.91

Species	White yaks	974	186	19.10
	Black yaks	610	148	24.26

Region	Tianzhu	974	186	19.10
	Luqu	146	33	22.60
	Maqu	464	115	24.78

Total		1584	334	21.09

**Table 2:** Odds ratios for specie as risk factor for bovine leukemia virus (BLV) seroprevalence in yaks (*n* = 1584).

Factor	Group	Prevalence (%)	OR	95% CI	*P* value
Specie	White yaks	19.10	Reference		
	Black yaks	24.27	1.36	1.06–1.73	0.014

**Table 3:** Seroprevalence of bovine leukemia virus (BLV) infection in yaks in different farms in Gansu province, northwestern China.

Region	Farm	No. tested	No. positive	Prevalence (%)
Tianzhu	1	220	51	23.18
	2	273	51	18.68
	3	231	44	19.05
	4	250	40	16.00

Maqu	5	81	19	23.46
	6	147	35	23.81
	7	103	29	28.16
	8	133	32	24.06

Luqu	9	60	15	25.00
	10	34	8	23.53
	11	52	10	19.23

Total		1584	334	21.09
